# Beam quality correction factors for dose measurements around ^192^Ir brachytherapy sources

**DOI:** 10.1002/acm2.14575

**Published:** 2024-11-27

**Authors:** Zoi Thrapsanioti, Vasiliki Peppa, Costas J. Hourdakis, Pantelis Karaiskos, Aristea Lekatou, Evaggelos Pantelis

**Affiliations:** ^1^ Medical Physics Laboratory Medical School National and Kapodistrian University of Athens Athens Greece; ^2^ Ionizing Radiation Unit Greek Atomic Energy Commission (EEAE) Agia Paraskevi Greece; ^3^ Radiotherapy department Alexandra Hospital Athens Greece

**Keywords:** beam quality correction factors, brachytherapy, dosimetry measurement, quality assurance

## Abstract

**Purpose:**

To provide beam quality correction factors ($k_{Q,Qo}$) for detectors used in ^192^Ir brachytherapy dosimetry measurements.

**Materials and Methods:**

Ten detectors were studied, including the PTW 30013 and Exrading12 Farmer large cavity chambers, seven medium (0.1–0.3 cm^3^) and small (< 0.1 cm^3^) cavity chambers, and a synthetic microdiamond detector. The k_Q,Qo_ correction factors were calculated at distances from 1 to 10 cm away from an ^192^Ir source, using the EGSnrc Monte Carlo (MC) code. All detectors were calibrated in a ^60^Co 10 × 10 cm^2^ reference field provided by standard calibration laboratories. The impact of the central electrode, stem and wall on the detectors’ responses in ^192^Ir photon energies was investigated. An experimental procedure was additionally applied for dose measurements around a microSelectron‐v2 ^192^Ir high dose rate (HDR) brachytherapy source using a motorized water phantom.

**Results:**

Farmer chambers underestimated the dose near the source due to signal volume averaging effects, resulting in *k_Q,Qo_* values ranging from 1.177 and 1.317 at 1 cm, decreasing with distance to between 0.980 and 1.005 at 10 cm. Small cavity volume detectors should be used close to the source. The *k_Q,Qo_* for the studied small and medium cavity volume detectors were found to be close to unity (within 1.3%), showing also a small dependence on source‐to‐detector distance, except for ion chambers containing high‐Z materials in their construction. The presence of high‐Z materials caused an overresponse in these detectors, resulting in *k_Q,Qo_* values ranging from 0.950 at 1 cm to 0.729 at 10 cm away from the source. A dose rate constant of (1.114 ± 0.023)cGyh^−1^U^−1^ was found in agreement with the literature (within 0.5%).

**Conclusions:**

*k_Q,Qo_* values were calculated for dose measurements around ^192^Ir brachytherapy sources. Farmer chambers should be preferred for measurements at increased distances, whereas small or medium cavity volume detectors, not containing high‐Z materials, should be used close to the source.

## 1 INTRODUCTION

In high dose rate (HDR) brachytherapy, doses greater than 5 Gy per fraction are delivered to well‐defined tumor volumes of the human body in a limited number of fractions (< 5).^1^, ^2^ Over the last decade brachytherapy has rapidly evolved, with significant advancements in equipment, physics, and clinical processes. These include the introduction of new equipment and applicators, the introduction of multimodality 3D imaging, patient‐specific optimization, and volume‐based dose prescription strategies.^2^, ^3^, ^4^, ^5^ Brachytherapy dose calculation algorithms have also evolved, shifting from standard water‐based dose cube superposition to advanced model‐based and deep convolutional network (CNN)‐based dose calculation algorithms.^6^, ^7^, ^8^ As the complexity of brachytherapy treatment procedures has increased, several sources of uncertainty have been recognized. The most common include confusion over units, incorrect afterloader‐catheter connections, incorrect dwell positions, and well‐type chamber calibration errors.^3^, ^4^, ^9^, ^10^, ^11^

Dosimetry audit (DA) is an important and useful tool in ensuring patient dosimetry accuracy and consistency. Existing brachytherapy DAs include verification of the reference air‐kerma rate (RAKR) of brachytherapy sources using well‐type ionization chambers,^11^ as well as dose verification at a single point (one dimension‐1D) close to the source (3–6 cm from the source center)^12^, ^13^, ^14^ or in two dimensions (2D) using radiochromic films and simple irradiation geometries.^15^, ^16^, ^17^, ^18^, ^19^, ^20^, ^21^, ^22^, ^23^ These services are offered either by post or on‐site visits. The dose measurements are usually performed either by passive dosimeters (e.g., TLD, OSLD, alanine, and radiochromic films) or ion chambers using simple geometric water, water equivalent, or plastic phantoms.

Dosimetry measurements around an ^192^Ir source situated in water require a dosimetry protocol to convert the measured signal to dose to water (*D_w_*). Currently, most standard laboratories do not provide absorbed *D_w_* calibration coefficients (*N_D,w_*) for ^192^Ir energies. Instead, they offer either *N_D,w_* coefficients for ^60^Co and higher energy photons, as well as air‐kerma calibration coefficients (*N_k_*) for ^60^Co and lower energy photons. Reynaert et al.^23^ suggested a protocol for measuring *D_w_* using Farmer chambers that is based on an interpolated *N_k_* (^192^Ir) calibration. The water‐to‐air mass energy absorption coefficient ratio, evaluated at ^192^Ir energy, along with Monte Carlo (MC) calculated corrections are used to convert the air‐kerma rate measured in water to *D_w_*. A more direct approach using dosimeters calibrated in ^60^Co beams and MC‐calculated beam quality correction factors ($k_{Q,Qo}$) has been also proposed.^12^, ^14^, ^22^ This approach, however, provided *k_Q,Qo_* values limited only for the used dosimeters, phantom materials, and dimensions. Chofor et al.^24^ suggested a semi‐analytical method for calculating *k_Q,Qo_* values for a selected list of dosimeters based on literature‐reported detector response versus energy data and mean photon energy values obtained through MC simulations.

In this work, MC methods were utilized to calculate *k_Q,Qo_* values for a number of ion chambers and a microdiamond detector calibrated in a ^60^Co reference beam as a function of distance away from an ^192^Ir source centered in a spherical water phantom. To resolve differences in the obtained *k_Q,Qo_* data, the effect of detectors’ stem, central electrode (if present), and wall on the *k_Q,Qo_* values was also studied. The calculated *k_Q,Qo_* data were used to perform ion chamber dosimetry measurements around an ^192^Ir HDR brachytherapy source positioned in a motorized water phantom.

## 2 MATERIALS AND METHODS

### 2.1 Dosimetric formalism

Assuming a dosimeter calibrated in a primary or secondary standard dosimetry laboratory (SSDL) using a reference photon beam quality *Q_o_* is available, conventional dosimetry protocols (e.g., TRS‐398^25^ or TG‐51^26^) can be employed to measure the dose around an ^192^Ir brachytherapy source situated in a water phantom, $D_{w,Q}$, according to:

(1) $$D_{w,Q}=M_{Q}N_{D,w,Q_{0}}k_{Q,Q_{0}}$$

where, *M_Q_* is the reading of the dosimeter in the experimental beam quality *Q* corrected for influence quantities (i.e., ion recombination, polarity, environmental conditions), $N_{D,w,Q_{0}}$ is the calibration coefficient of the dosimeter in terms of absorbed *D_w_* in the reference beam quality *Q*_o_ (commonly a ^60^Co photon beam). The beam quality correction factor, *k_Q,Qo_*, accounts for differences in the dosimeter's response in the reference and the experimental beam quality and can be obtained from:

(2) $$k_{Q,Qo}=\frac{{\left[D_{w}/{\bar{D}}_{\det}\right]}_{Q}}{{\left[D_{w}/{\bar{D}}_{\det}\right]}_{Q_{o}}}\;\frac{R_{Q_{o}}}{R_{Q}}$$

where, ${\bar{D}}_{\det,Q}$ and ${\bar{D}}_{\det,Q_{o}}$ is the absorbed dose to the active volume of the dosimeter in the experimental and the reference beam quality, respectively. $D_{w,Q}$ and $D_{w,Q_{o}}$ is the absorbed *D_w_* at the measurement point for the experimental and reference beam qualities, respectively. $R_{Q}=M_{Q}/{\bar{D}}_{\det,Q}$ (and similarly $R_{Q_{o}}$) is the intrinsic energy response of the dosimeters describing the generation of the physical signal and its collection efficiency within the dosimeter.^22^ Assuming that the intrinsic energy response of the studied dosimeters is constant (i.e., $R_{Q_{o}}/R_{Q}\;$= 1), the k*_Q,Qo_* can be determined using Equation (2). MC methods can be used to calculate the required dosimetric quantities, that is, ${\bar{D}}_{\det,Q}$, ${\bar{D}}_{\det,Q_{o}}$, $D_{w,Q}$, and $D_{w,Q_{o}}$ (see Section 2.3).

### 2.2 Details of studied dosimeters

Nine ion chambers and a solid‐state detector were investigated in this study. The geometrical and constructional details of the studied dosimeters are presented in Table 1. The studied solid‐state detector was the PTW 60019 synthetic microdiamond (PTW Freiburg GmbH, Germany) having a sensitive volume of 4 × 10^−3^ mm^3^ (diameter 2.2 mm, thickness 4 µm). While this detector has been designed for small field dosimetry measurements, it has been also used for quality assurance dose measurements in brachytherapy applications.^22^ The studied ion chambers were classified into three categories based on their cavity volumes: small (less than 100 mm^3^), medium (100–300 mm^3^), and large (greater than 300 mm^3^). The IBA CC003 chamber (IBA Dosimetry GmbH, Germany) had the smallest cavity volume, measuring 3 mm^3^, among the studied chambers. The specific chamber features a spherical cavity with a diameter of 2 mm and a central electrode with a diameter of 1 mm. Besides the CC003, the small cavity volume chambers studied included the PTW 31014 and 31016, the IBA CC01G, and the Extradin A16 (Standard Imaging Inc, USA) chambers (see Table 1). The two medium cavity volume chambers studied were the PTW 31010 with a volume of 125 mm^3^ and the PTW 31013 with a volume of 300 mm^3^. The large cavity volume chambers consisted of the PTW 31013 Farmer‐type chamber with a volume of 600 mm^3^ and the Exradin A12 Farmer‐type chamber with a volume of 640 mm^3^ featuring 25.8 mm and 6.1 mm, cavity length, and diameter respectively. The central electrodes of the studied ion chambers are made of aluminum (PTW) and graphite (IBA). The studied Exradin chambers had central electrodes made of C552 (A12) and silver‐plated copper‐covered steel (A16). The wall of the PTW chambers is made of graphite and polymethylmethacrylate (PMMA), whereas in the IBA and Exradin chambers it is made of C552.

**TABLE 1:** Geometrical and constructional details of studied dosimeters.

		Active volume		Wall		Electrode	
Detector	Cavity volume	diameter	Length	material	Thickness	material	thickness
PTW 31014	15	2.0	5.0	Gr/PMMA^a^	0.09/0.57	Al	0.3
PTW 31016	16	2.9	2.9	Gr/PMMA	0.09/0.57	Al	0.3
PTW 31010	125	5.5	6.5	Gr/PMMA	0.15/0.55	Al	1.1
PTW 31013	300	5.5	16.25	Gr/PMMA	0.15/0.55	Al	0.9
PTW 30013	600	5.5	23	Gr/PMMA	0.09/0.34	Al	1.1
PTW 60019	4 × 10^−3^	2.2	10^−3^	Diamond/Epoxy Resin	0.3/0.614	Al	0.01
IBA CC003	3	2.0^b^	2.0	C552	0.5	Gr	1.0^b^
IBA CC01G	10	2.0	3.6	C552	0.5	Gr	0.6
Exradin A12	640	6.1	25.8	C552	0.5	C552	1.0
Exradin A16	7	3.4	2.5	C552	0.5	SPC^c^	0.3

*Note*: All dimensions are expressed in mm. Detectors’ cavity volumes are expressed in mm^3^.

Abbreviations: Gr/PMMA, Graphite/Polymethylmethacrylate; SPC, Silver‐plated copper‐covered steel.

^a^ Gr/PMMA.

^b^ The active volume and the central electrode of this chamber are spherical.

^c^ SPC.

### 2.3 MC calculations

**FIGURE 1:**
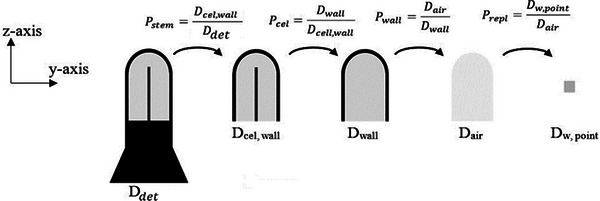
Illustration of the detector models simulated to calculate the dose to the sensitive volume of each detector (*D*_det_, *D*_cel,wall_, *D*_wall_, *D*_air_) and the corresponding perturbation factors shown in the dose ratios. The *y–z* plane of the used coordinate system is depicted, indicating the orientation of all detectors except for the microdiamond (PTW 60019). The ^192^Ir source was positioned at the origin of the coordinate system. The microdiamond was simulated with its sensitive volume facing the source and the stem aligned with the *y*‐axis.

The EGSnrc MC code^27^ was used to obtain the ratios ${\left[D_{w}/{\bar{D}}_{\det}\right]}_{Q}$ and ${\left[D_{w}/{\bar{D}}_{\det}\right]}_{Q_{o}}$ included in Equation (2) and calculate the *k_Q,Qo_* of the studied dosimeters. It is noted that EGSnrc has been proven accurate in predicting detector dose response in a wide range of radiation beam qualities including ^192^Ir photon beam energies^28^, ^29^, ^30^ and has been used extensively in the peer review literature in providing detector‐specific correction factors for dosimetry measurements in radiotherapy applications.^31^, ^32^, ^33^ All phantom, source, and detector geometries were modeled using the egs++ geometry library available with the EGSnrc code. To improve calculation efficiency, the ^192^Ir source was modeled as an isotropic point source centered within a spherical water phantom with a diameter of 30 cm. Photon energy was sampled from the fluence spectrum emerging an encapsulated microSelectron HDR (mHDR) ^192^Ir source (Elekta AB, Sweden).^34^ Geometry and material composition of the studied dosimeters were modeled based on corresponding blueprints provided by the manufacturers and/or found in the literature. All dosimeters were modeled with the geometric center of their sensitive volume lying on the transverse source bisector (*y*‐axis) and with their stem parallel to the source longitudinal axis (*z*‐axis) (see Figure 1), except for the 60019 microdiamond which was simulated with its stem parallel to *y*‐axis and its active volume facing the source. Dosimetry calculations were performed with each dosimeter situated at *y*‐values spanning from 1 cm up to 10 cm with a step of 1 cm. A small cubic water voxel of 0.5 mm side was used to calculate the *D_w_* at the same positions on the *y*‐axis with dosimeters removed.

Reference beam quality calculations were performed using setups equivalent to those employed for the calibration of the dosimeters in a primary or secondary dosimetry standard laboratory. These setups included a source to detector distance of 100 cm, a field size of (10 × 10) cm^2^, water phantom dimensions of (30 × 30 × 30) cm^3^, and a dose calculation depth of 5 cm. The reference beam was modeled as a ^60^Co point source, with the photon spectrum taken from the work of Mora et al.^35^ All detectors were modeled with their stem perpendicular to the beam axis except for the microdiamond, which was simulated with its stem parallel to the beam axis due to its design.

The egs_chamber user code was used for all dosimetry calculations.^30^ The default MC transport parameters were used along with the XCOM photon cross‐sections. The cutoff energies and production thresholds were set to 512 keV for electrons and 1 keV for photons. Stopping power data for all materials were obtained taken from ICRP report 49^36^ except for the graphite, air, and liquid water, which were taken from ICRU report 90.^37^ Intermediate phase space scoring (IPSS) and photon cross section enhancement (XCSE) variance reduction techniques were employed to increase simulation efficiency. The default values were used for the remaining EGSnrc transport parameters and cross‐sectional options. Electron transport was performed using the PRESTA‐II algorithm, with the EXACT boundary cross algorithm and an ESTEPE value of 0.25.

Attention was given to the effect of the constructional materials and geometrical characteristics of the studied detectors on their dose response. Therefore, a decomposition technique^31^, ^38^ was followed to calculate the effect of the stem, central electrode, and wall materials of each detector on its dose response. The effective replacement of the detector's sensitive volume with a small water voxel was also studied. All these factors, known as perturbation factors (*p*_cel_, *p*_stem_, *p*_wall,_ and *p*_repl_),^25^, ^26^, ^39^ are represented by ratios of doses scored in two different scoring volumes, each one of them describing the effect examined. As shown in Figure 1, *p*_stem_ was defined as the ratio of the dose to detector's sensitive volume without and with the stem simulated, *p*_cel_ as the ratio of the dose to detector's sensitive volume without and with the central electrode simulated, *p*_wall_ as the ratio of the dose to detector's sensitive volume without and with the wall simulated and *p*_repl_ as the ratio of the dose to a small voxel of water to the dose to detector's sensitive volume. It is noted that the microdiamond detector electrodes were not modeled and the diamond base where the sensitive volume is embedded was assumed as the “stem”, while the rest of the materials surrounding the sensitive volume (epoxy resin, RW3, etc.) were considered as a wall.

### 2.4 Dosimetry measurements around an ^192^Ir HDR source

**FIGURE 2:**
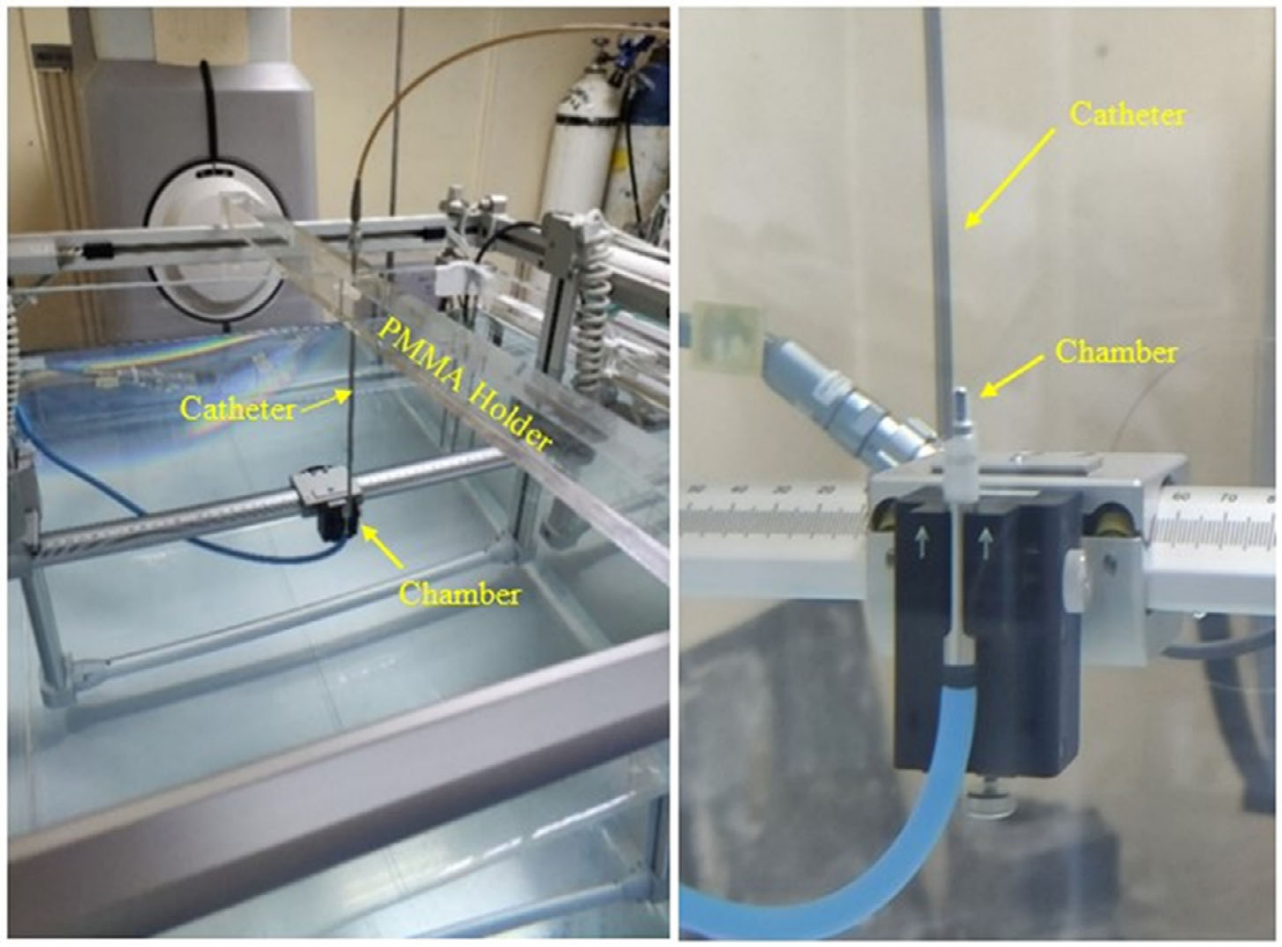
The experimental setup used for dosimetry measurements around the micorSelectron‐v2 ^192^Ir HDR source. HDR, high dose rate.

*D_w_* measurements was performed for an ^192^Ir mHDR‐v2 brachytherapy source positioned inside a motorized water phantom of (67.5 × 67.5 × 56) cm^3^ external dimensions (position resolution = 0.1 mm, position accuracy = ± 0.1 mm, Blue Phantom2, IBA Dosimetry GmbH, Germany) (see Figure 2). An appropriate PMMA holder was constructed to align vertically a metallic brachytherapy catheter used to guide the source at the irradiation position (Figure 2). The catheter used was an intrauterine straight tube of the Nucletron Miami Vaginal Applicator, which is made of stainless steel. It has an external lower diameter ‐ending to the tip‐ of 3 mm and a length of 10 cm, whilst the inner upper diameter is 4 mm and the respective length is 17.8 mm. The catheter ensured also that the longitudinal axis of the source was parallel to the dosimeter's stem during irradiation and that the source's center during irradiation was situated at a depth of 15 cm beneath the water surface. Dosimetry measurements were performed using the PTW 31016 ion chamber. With the source at the irradiation position, a scanning technique was employed to align the ion chamber with the source and define the coordinate system for reporting measured dose values with regard to the source center.

The air‐kerma strength, *S_k_*, of the source on the day of irradiation, was equal to 21954.50 U. After detector alignment, and with the source in irradiation position, the detector was instructed to move 1 cm away from the source along the source's transversal bisector and integrate the collected charge for 5 min. Measurements were repeated four times for statistical reasons. After correcting for environmental conditions, polarity, and ion‐recombination effects, Equation (1) was used to calculate *D_w_*. Dose away and along profile measurements were also acquired for away and along distances up to 3 cm from the source. The measured charge was integrated for variable times ranging from 2 min at points close to the source up to 10 min for points lying away from the source.

### 2.5 Uncertainty budget

All MC calculations were performed by a super‐computer consisting of 426 computational nodes with ten Ivy Bridge Intel Xeon E5 v2 processors per node, which offered a total of 8520 CPU cores (computational threads) clocked at 2.8 GHz.^40^ A total number of up to 10^10^ photon histories were simulated, resulting in a statistical uncertainty of the calculated dose results equal to 0.1% near the source increasing to 0.5% at 10 cm away from the source. Statistical uncertainties of the calculated *k_Q,Qo_* correction factors ranged between 0.2% at 1 cm increasing to 0.7% at 10 cm away from the ^192^Ir source. The uncertainty owing to the simulation geometry, radiation sources, transport options, and interaction cross sections used in the EGSnrc MC simulations as well as the construction details of the studied detectors was taken equal to 0.78%.^41^

Experimental uncertainties were evaluated following the recommendations of the Guide to the Expression of Uncertainty in Measurement.^42^ The type A (statistical) uncertainty component was estimated as the standard deviation of the mean dosimeter reading (0.01%). The type B (nonstatistical) component was assessed by considering uncertainties related to the positioning of the detector and source, the calibration coefficient of the detector (0.55%), corrections to the measured signal for environmental conditions, ion recombination, polarity, and electrometer (0.1%), and the beam quality correction factor. An analysis of the uncertainty budget of the measured dose at the reference away distance from the source (i.e., *y* = 1 cm) is given in Table 2. The major contribution to the combined dose uncertainty of 1.55% (*k* = 1) comes from the source to detector positioning uncertainty. This uncertainty was estimated as the standard deviation of the mean of the measured signal of five measurements where both the source and the chamber were repositioned between each measurement. For the 1 cm distance away from the source the positioning uncertainty was estimated equal to 1.2%. It is noted that positioning uncertainty decreases as distance from the source increases. A positioning uncertainty of 0.2% has been estimated at a 6 cm away distance using published dose away and along data for the used HDR source.^43^

**TABLE 2:** Uncertainty budget for the PTW 31016 ion chamber dosimetry measurements performed in a water phantom using the mHDR‐v2 ^192^Ir brachytherapy source.

Uncertainty component	Type A (%)	Type B (%)
$k_{Q,Qo}$	0.21	0.78^41^
$N_{D,w,Q_{0}}$		0.55
$p_{\mathrm{ion}}p_{\mathrm{pol}}p_{\mathrm{elec}}p_{\mathrm{TP}}$		0.10
Signal measurement	0.01	
Source—detector positioning		1.2
Combined dose uncertainty (*k* = 1)	1.55	

*Note*: Presented uncertainty values correspond to an away distance of 1 cm.

## 3 RESULTS

### 3.1 Beam quality correction factors, *k_Q,Qo_*

**FIGURE 3:**
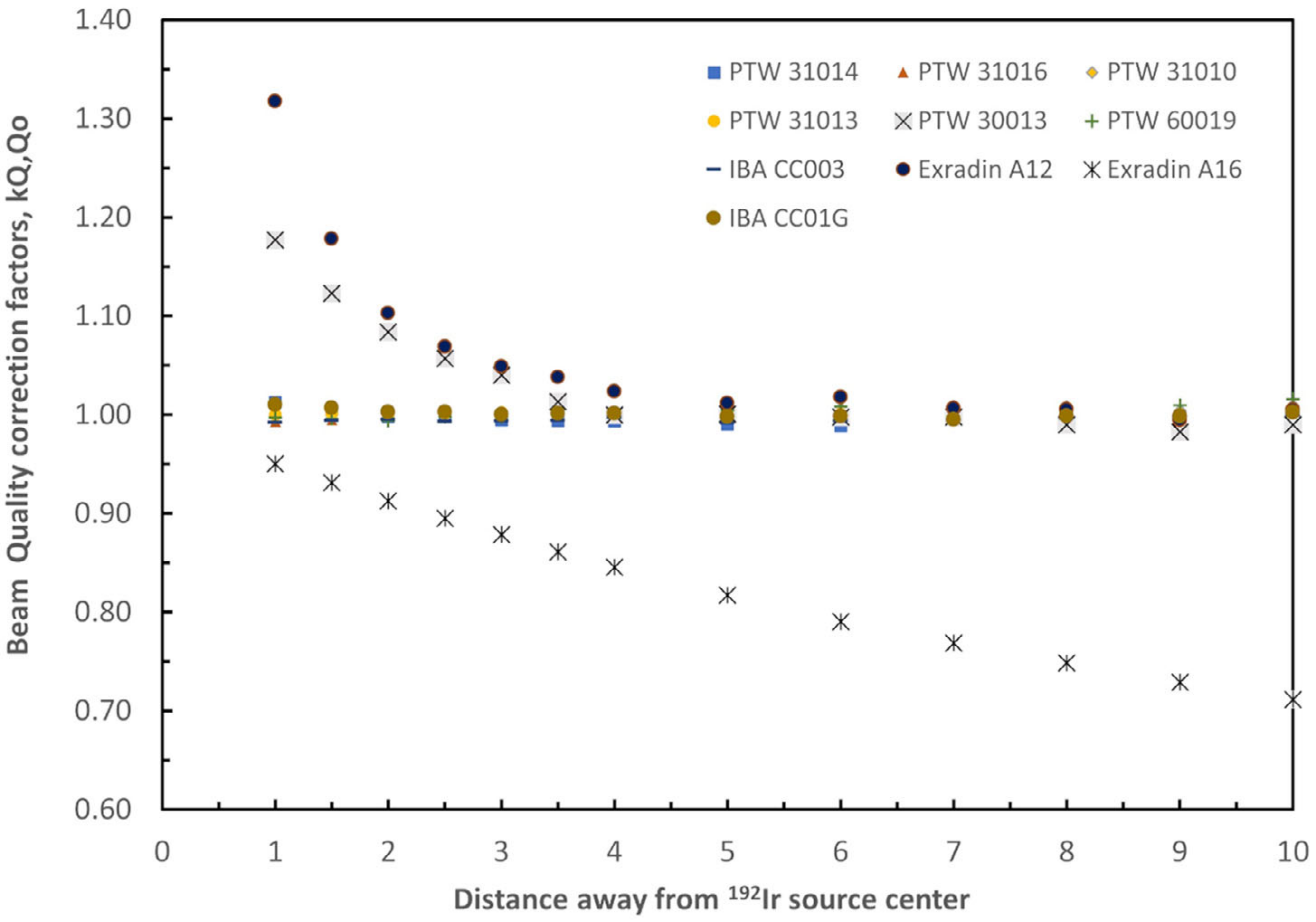
Beam quality correction factors, *k_Q,Qo_*, for the studied detectors plotted as function of distance away from an ^192^Ir source centered within a spherical water phantom with a diameter of 30 cm.

The calculated *k_Q,Qo_* beam quality correction factors of the studied detectors are plotted in Figure 3 as a function of distance away from the ^192^Ir source and given numerically in Table 3. As can be seen among the studied detectors A16 small cavity volume chamber, and the large cavity volume 30013 and A12 Farmer chambers present the largest dependence with distance away from the source. The *k_Q,Qo_* for the A16 was found equal to 0.950 at *y* = 1 cm decreasing with distance away from the source and reaching a value of 0.711 at *y* = 10 cm. On the contrary, the *k_Q,Qo_* for the 30013 and the A12 chambers were found equal to 1.177 and 1.317 at *y* = 1 cm, respectively. As distance away increases the *k_Q,Qo_* for the 30013 and the A12 reduce by 0.980 and 1.005 at *y* = 10 cm, respectively. The k*_Q,Qo_* for the rest of the studied detectors were found close to unity for the studied away distances (within 1.3%).

**TABLE 3:** Beam quality correction factors, k*_Q,Qo_*, for the PTW 31014, 31016, 31010, 31013, 30013, and 60019, the IBA CC003 and CC01G, and the Exradin A12 and A16 detectors.

	PTW					
Distance from source (cm)	31014	31016	31010	31013	30013	60019
1	1.013 (3)	0.993 (3)	0.998 (2)	1.000 (2)	1.177 (3)	0.997 (2)
1.5	1.005 (3)	0.995 (3)	1.000 (2)	1.001 (2)	1.123 (3)	0.996 (2)
2	0.998 (3)	1.000 (3)	1.002 (2)	1.000 (2)	1.084 (3)	0.994 (2)
2.5	0.999 (3)	0.998 (3)	1.000 (2)	1.001 (2)	1.057 (3)	0.998 (2)
3	0.994 (3)	1.000 (3)	1.002 (2)	1.002 (3)	1.040 (3)	0.998 (3)
3.5	0.994 (3)	1.000 (4)	1.001 (3)	1.001 (3)	1.013 (3)	1.000 (3)
4	0.993 (4)	0.998 (4)	1.001 (3)	1.000 (3)	1.000 (3)	1.002 (3)
5	0.990 (4)	1.001 (4)	1.001 (3)	1.002 (3)	1.001 (3)	1.004 (3)
6	0.988 (4)	1.003 (4)	1.007 (4)	1.002 (4)	0.998 (4)	1.009 (4)
7	0.995 (5)	1.005 (5)	0.999 (4)	1.000 (4)	0.997 (4)	1.008 (4)
8	0.991 (5)	1.004 (5)	1.008 (5)	0.999 (4)	0.990 (4)	1.001 (4)
9	0.997 (5)	1.00 7 (5)	1.004 (5)	1.000 (5)	0.983 (5)	1.009 (5)
10	1.003 (5)	0.997 (5)	1.009 (5)	1.001 (5)	0.980 (5)	1.016 (5)

	IBA		Standard imaging	
Distance from source (cm)	CC003	CC01G	Exradin A12	Exradin A16
1	0.992 (5)	1.010 (3)	1.317 (3)	0.950 (3)
1.5	0.994 (5)	1.007 (3)	1.178 (3)	0.931 (3)
2	0.995 (5)	1.003 (3)	1.103 (3)	0.912 (3)
2.5	0.993 (5)	1.003 (3)	1.069 (3)	0.895 (3)
3	0.994 (5)	1.000 (3)	1.049 (3)	0.878 (3)
3.5	0.994 (5)	1.001 (3)	1.038 (3)	0.861 (3)
4	0.997 (5)	1.001 (4)	1.024 (3)	0.845 (3)
5	0.991 (5)	0.998 (4)	1.011 (4)	0.817 (3)
6	0.993 (5)	0.999 (4)	1.018 (4)	0.790 (4)
7	0.995 (6)	0.995 (5)	1.007 (4)	0.768 (4)
8	0.996 (6)	0.998 (5)	1.005 (5)	0.749 (4)
9	0.998 (6)	0.999 (5)	0.994 (5)	0.729 (4)
10	0.999 (6)	1.002 (5)	1.005 (6)	0.711 (5)

### 3.2 Detector response perturbation factors

**FIGURE 4:**
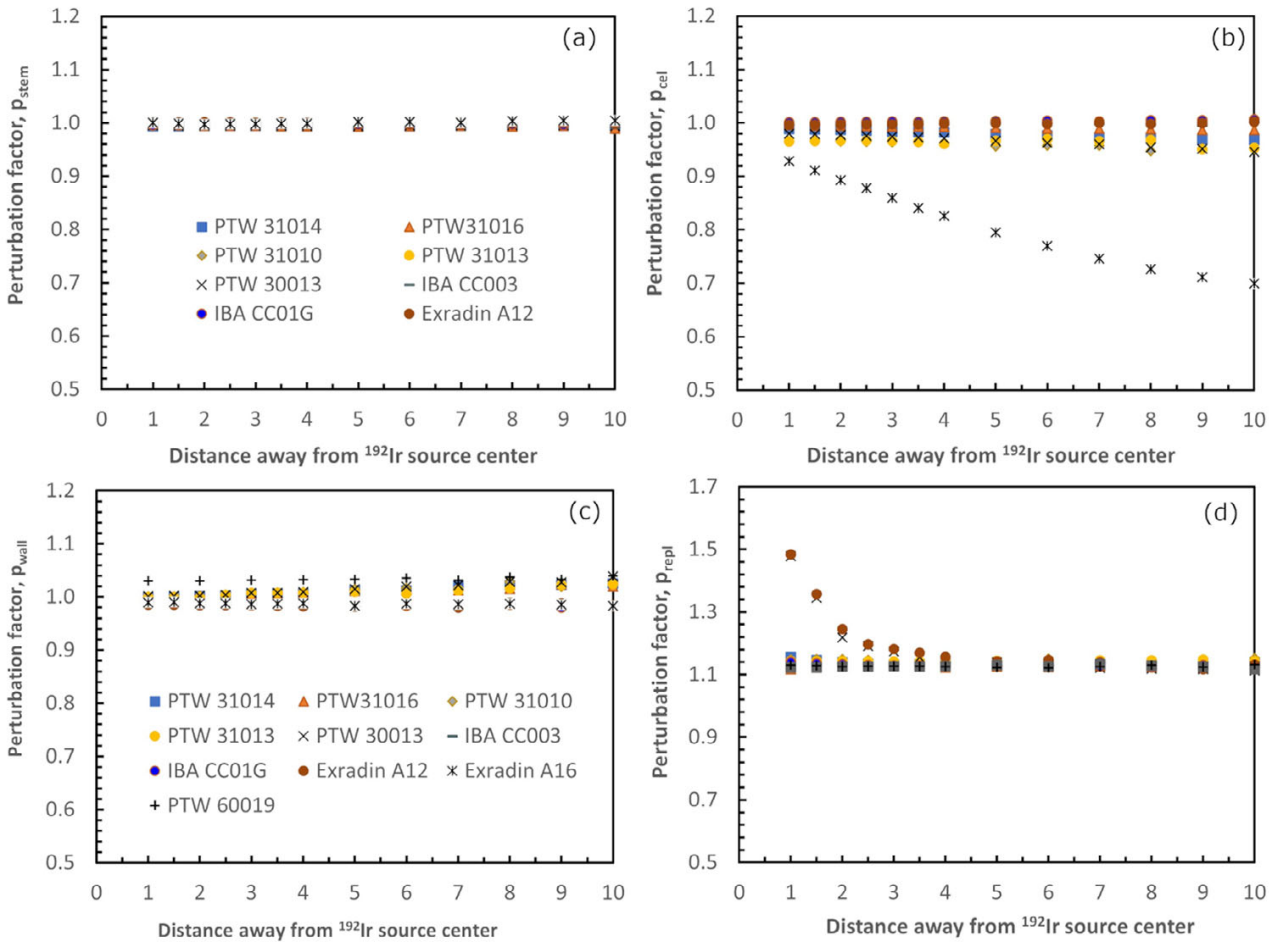
Stem (a), central electrode (b), wall (c), and replacement (d) perturbation factors for the studied detectors as a function of distance away from an ^192^Ir brachytherapy source. For the PTW 60019 microdiamond the central electrode is not defined, the diamond base where the sensitive volume is embedded was considered as “stem” and the surrounding materials of the sensitive volume as “wall”.

Further insight into the factors affecting the dose response of the studied detectors is given in Figure 4 where the *p*_stem_, *p*_cel_, *p*_wall,_ and *p*_repl_ perturbation factors are plotted as a function of distance away from the source. As can be seen in Figure 4, the stem affects the response of the studied ion chambers less than 0.7% being constant within uncertainties throughout the studied distance range. For the 60019 detectors, the diamond base (assumed to be expressed by *p*_stem_) is found to affect the absorbed dose by 1.2% at *y* = 1 cm reducing to less than 0.2% at greater *y*‐values.

The central electrode was found to increase the response of the A16 ion chamber, showing a *p*_cel_ factor of 0.928 at *y* = 1 cm. This increase is further pronounced with distance away from the source with *p*_cel_ decreasing to 0.699 at *y* = 10 cm. Moreover, to a smaller degree, the central electrode was found to affect in a similar manner the response of the 31014, 31016, 30013, 31010, and 31013 ion chambers. In more detail, the *p*_cel_ of the 31014 ion chamber was found equal to 0.989 at *y* = 1 cm decreasing to 0.968 at *y* = 10 cm. For the 31 016 ion chamber, the *p*_cel_ was found equal to 0.996 at *y* = 1 cm, slightly decreasing to 0.987 at *y* = 10 cm. For the 31 010 and 31 013 ion chambers, the same *p*_cel_ value of 0.966 was calculated at *y* = 1 cm, which decreased with distance away from the source reaching 0.952 and 0.953, respectively, at *y* = 10 cm. For the 30 013 Farmer chamber, the *p*_cel_ was found equal to 0.980 at *y* = 1 cm, decreasing to 0.946 at *y* = 10 cm. On the contrary, the effect of the central electrode was found to minimally affect (less than 0.5%) the response of the CC01G, CC003, and A12 ion chambers throughout the studied distance range.

The presence of the wall was found to decrease by less than 1% the response of the studied ion chambers at points close to the source. As the distance from the source increases; however, the wall was found to reduce the dose to the air cavity (i.e., *p*_wall_ greater than unity) for the PTW ion chambers all having the same wall material. This effect is associated with *p*_wall_ factors of 1.025 for the 31014 and 31016 ion chambers, 1.027 for the 31010 and 31013, and 1.039 for the 30013 at *y* = 10 cm. The wall for the studied Exradin and IBA ion chambers was found to increase the dose to the sensitive volume by about 1% throughout the studied distance range. As far as the 60019 microdiamond is concerned, the wall was found to decrease the dose deposited in the sensitive volume resulting in *p*_wall_ factors equal to 1.030 at *y* = 1 cm, which increased with distance from the source reaching up to 1.040 at *y* = 10 cm.

Signal volume averaging effects, fluence perturbation due to the presence of the detector's sensitive volume in the radiation field, and differences in the electronic stopping power ratios are included in the *p*_repl_ perturbation factor. As can be seen in Figure 4, Farmer ion chambers were found to have the largest *p*_repl_ values among the studied detectors and distance range. In detail, for the A12 the *p*_repl_ was found equal to 1.484 at *y* = 1 cm, which decreased with distance away from the source reaching a value of 1.125 at *y* = 10 cm. Similarly, for the 30 013 *p*_repl_ values of 1.478 and 1.120 were found at *y* = 1 and 10 cm, respectively. The *p*_repl_ for the 31014 was found equal to 1.155 at *y* = 1 cm and 1.126 for *y*‐values ≥ 3 cm. For the 31016, the *p*_repl_ was found equal to 1.118 at *y* = 1 cm increasing to 1.126 for *y* ≥ 2 cm, whereas for the 31010, 31013, and A16 chambers *p*_repl_ was found equal to 1.142, 1.144, and 1.128, respectively, for the studied distance range away from the source. Concerning the CC01G and CC003 chambers, the *p*_repl_ was found equal to 1.143 and 1.131 at *y* = 1 cm, respectively. A small dependence with distance was observed with *p*_repl_ at *y* = 10 cm, found equal to 1.124 for the CC01G and 1.138 for the CC003. Finally, for the PTW 60019 microdiamond, the *p*_repl_ was equal to 1.127 for the studied distance range away from the source.

### 3.3 Experimental dosimetry

Measured dose rate values around the mHDR‐v2^192^Ir brachytherapy source are presented in Table 4. In the same table, the corresponding dose data exported from the treatment planning system (TPS) are also included for reasons of comparison. The presented data show a good agreement within uncertainty between the measured and the TPS dose values at all points except those situated at *z* = −2 cm and *z* = −3 cm. At these points, the measured dose values are up to 4.7% lower than the corresponding TPS results. Based on the measured dose rate value at *y* = 1 cm and the air‐kerma strength of the source (*S_K_* = 21954.50 ± 1.7% (*k* = 1) U) on the day of irradiation, the dose rate constant of the mHDR‐v2 source was calculated and found equal to (1.114 ± 0.023) cGy h^−1^ U^−1^.

**TABLE 4:** Measured and treatment planning calculated dose rate values at points along *y*‐axis (source transversal bisector) and along *z*‐axis (source longitudinal axis) at *y* = 1 cm for the mHDR‐v2 ^192^Ir brachytherapy source.

*y* coordinate (cm)	Measured dose rate (Gy/min)	TG‐43 dose rate (Gy/min)	$\frac{{\dot{D}}_{\mathrm{meas}}-{\dot{D}}_{\mathrm{TG}43\;}}{{\dot{D}}_{\mathrm{TG}43\;}}$ (%)
1	4.415	4.059	0.5
1.5	1.831	1.820	0.6
2	1.034	1.028	0.5
3	0.453	0.459	−1.2

***z* coordinate (cm)**			
−3	0.335	0.352	−4.7
−2	0.711	0.744	−4.4
−1	1.978	1.987	−0.5
0	4.415	4.059	0.5
1	1.971	1.984	−0.6
2	0.735	0.745	−1.4
3	0.355	0.354	0.3

Abbreviations: HDR, HDR, high dose rate; mHDR, microSelectron HDR.

## 4 DISCUSSION

Beam quality correction factors, *k_Q,Qo,_* were calculated for a series of nine ion chambers with a wide variety of cavity volumes and construction materials and a solid‐state detector, as a function of distance away from an ^192^Ir brachytherapy source. Provided that the studied detectors are calibrated in a ^60^Co reference field, these values can be used to account for differences in their dose response between the reference and ^192^Ir photon beam qualities. Farmer‐type ion chambers were found to require increased correction factors, when used to measure the dose in the vicinity of ^192^Ir sources (i.e., at *y* ≤ 5 cm). These correction factors were found to reach up to 1.177 for the 30013 and 1.317 for the A12 Farmer chamber at *y* = 1 cm. These values are attributed to the large cavity volumes of these chambers (see Table 1), which results in increased signal‐averaging effects. As the distance from the source increases, signal volume averaging effects reduce and the *k_Q,Qo_* correction factors decrease reaching at *y* = 10 cm a value of 0.980 for the 30 013 and 1.005 for the A12. The calculated *k_Q,Qo_* correction factors for the 30013 agree within uncertainties with the corresponding values of 1.174 at *y* = 1.5 cm and 0.985 at *y* = 10 cm reported by Araki et al.^13^ Moreover, it should be noted, that at points lying at increased distances from the source, photon fluence is substantially decreased due to the inverse square law and Farmer chambers characterized by increased sensitivity is preferred for corresponding dosimetry measurements.^13^, ^44^

Close to brachytherapy sources, small or medium cavity volume detectors should be used to reduce volume averaging effects. For the small and medium cavity ion chambers studied herein the *k_Q,Qo_* correction factors were found to deviate less than 1.3% from unity over the investigated distance range. The exception to this finding is the *k_Q,Qo_* correction factor for the A16 chamber. For this detector, an increased dependence with distance from the source was observed, with the *k_Q,Qo_* values found equal to 0.950 at *y* = 1 cm and 0.711 at *y* = 10 cm. Using a decomposition technique, it was found that the A16 chamber overresponds in ^192^Ir photon energies due to high‐Z materials used for manufacturing its central electrode (Table 1). The presence of these high‐Z materials increases the probability of photon interactions with the electrode, which results in more electrons being set in motion, and consequently, a higher measured dose at low energies compared to the reference beam quality. Moreover, as the distance from the source increases, the mean photon energy decreases,^24^, ^45^ further enhancing the interaction probability of photons with the central electrode and the observed overesponse.^46^ For the rest of the studied ion chambers, the central electrode is composed of low‐Z materials, affecting their dose response by less than 2%, also depending on the distance from the source. This dependence, albeit to a small degree, could be attributed to the softening of the photon energy spectrum as the distance from the source increases. A similar behavior was also found for the stem and wall, both made of low‐Z materials in all studied detectors. The above findings suggest that high‐Z materials should be avoided in manufacturing ion chambers for dosimetry measurements in low‐energy photon beams.

Muir et al.^46^ used MC methods to calculate the *p*_cel_ of an extended series of ion chambers to be used for dosimetry measurements in low and high photon beam energies including ^192^Ir photon spectrum. For the A16 ion chamber, a *p*_cel_ perturbation factor of about 0.93 was reported at 1 cm away from the source, which decreased to 0.77 at 8 cm away from the source. These values are in close agreement with the corresponding results of 0.928 at *y* = 1 cm and 0.726 at *y* = 8 cm calculated herein. The increased deviation observed at points away from the source is attributed to differences in the dimensions of the used water phantoms (30 cm in diameter used herein vs. [50 × 50 × 50] cm^3^ used by Muir et al.^46^), affecting the photon spectrum close to the edges of the phantom.^24^ Other sources of uncertainty involve differences between the ^192^Ir spectrum emitted by the mHDR source used in this study and the VariSource (Varian Medical Systems Inc., USA) HDR source used by Muir et al.,^46^ as well as in differences between the used A16 ion chamber simulation models.

Chofor et al.^24^ suggested a semiempirical method based on published dose response functions of a variety of ion chamber and solid state detectors, and MC calculated mean photon energy values as a function of distance away from ^192^Ir sources. Using the semiempirical method and the data provided by Chofor et al.^24^ a *k_Q,Qo_* of 1.017 was calculated for the 31014 at *y* = 1 cm. This value is in excellent agreement (within 0.4%) with the corresponding *k_Q,Qo_* value of 1.013 ± 0.003 reported in this study for the same ion chamber and distance away from the source. Moreover, a good agreement was observed between the calculated *k_Q,Qo_* values reported in this study and those obtained using the method of Chofor et al.^24^ for the 31 014 and for points further away from the source (the largest deviation was found equal to 1.9% at *y* = 5 cm).

The *k_Q,Qo_* correction factors for the 60 019 microdiamond were found to vary slightly with distance away from the source, taking values of 0.997 at *y* = 1 cm and 1.016 at *y* = 10 cm. The small sensitive volume and the low‐Z materials used in the construction of the specific detector explain its similar dose response in ^60^Co and ^192^Ir photon energies. Kaveckyte et al.^22^ used the 60019 detector for dosimetry quality assurance measurements in brachytherapy applications using a mHDR‐v1 source. MC methods were used to calculate the *k_Q,Qo_* correction factor at various distances away from the brachytherapy source. *k_Q,Qo_* correction factor values of 1.003, 1.005, and 1.013 were reported at points lying 1.5, 2.5, and 5.5 cm away from the source, which is in excellent agreement with the corresponding data calculated in our study.

An experimental set‐up was used for *D_w_* measurements around the ^192^Ir mHDR‐v2 source. The setup used involved positioning the 31016 ion chamber with its stem parallel to the source's longitudinal axis inside a motorized water phantom. The symmetry of the dose distribution around the brachytherapy source was used along with a scanning technique to align the chamber at the measurement point. Dosimetry results were compared with the corresponding AAPM TG‐43 based results extracted from the TPS. Using the measured dose at *y* = 1 cm a dose rate constant of (1.114 ± 0.023) cGyh^−1^U^−1^ was found, which agrees within 0.5% with the corresponding consensus value of 1.109 cGyh^−1^U^−1^ reported by Perez‐Calatayud et al.^47^ For the rest of the measurement points an agreement within 4.7% compared to the corresponding TPS dose values was observed.

Accurate positioning of the used detector around the brachytherapy source poses an increased challenge in corresponding dosimetry measurements. Araki et al.^12^, ^13^ constructed a water phantom containing two ion chambers positioned with their stem parallel to the longitudinal axis of an ^192^Ir brachytherapy source in a so‐called sandwich assembly. Using the specific setup and by taking the average reading of the two chambers, dose errors due to difficulties in absolute centering of the source in between the chambers were canceled to the first order. The authors applied their technique to measure the dose at *y* = 5 cm away from the mHDR‐v2 source and found an agreement with the corresponding TG‐43 data better than 1.3% and 0.73% using an A1SL and a 30013 ion chamber, respectively. The overall accuracy of the Araki et al.^12^, ^13^ method was estimated to be equal to 1.7% and 0.68%, for the A1SL and 30013 chambers, respectively. Sarfehnia et al.,^14^ used Gafchromic film and ionometric calibration procedures to determine the dose rate to water for HDR ^192^Ir brachytherapy sources. The results were compared to TG‐43‐based dose data and absolute dose measurements from a water calorimetry‐based primary standard. The accuracy of the detector setup was assured using a traveling microscope. Based on water calorimetry, the authors measured over a 3‐year period a dose rate to water at 5.5 cm away from different mHDR sources of (361 ± 7) µGyh^−1^U^−1^. Moreover, the dose rate normalized to air‐kerma strength for the used techniques was found to agree with the water calorimetry results to within 0.83%. An overall 1‐sigma uncertainty for ionization chamber dosimetry of 1.44% was reported, which is close to the uncertainty reported herein.

## 5 CONCLUSIONS

*k_Q,Qo_* beam quality correction factors were calculated for nine ion chambers with cavity volumes spanning from 3 mm^3^ to 640 mm^3^ and for a solid‐state detector, to be used for *D_w_* measurements around ^192^Ir sources. The *k_Q,Qo_* correction factor for the 30 013 and A12 Farmer chambers were found equal to 1.177 and 1.317, respectively at *y* = 1 cm. These *k_Q,Qo_* values are mainly attributed to signal‐averaging effects. At increased distances from the source, the calculated *k_Q,Qo_* values decrease reaching a value of 0.980 and 1.005 for the 30 013 and A12 chambers, respectively, at *y* = 10 cm. Ion chambers made of high‐Z materials (e.g., A16 chamber) should not be used for dosimetry measurements in ^192^Ir photon energies.

## AUTHOR CONTRIBUTIONS

**Zoi Thrapsanioti**: Data collection; figure and table preparation; manuscript writing. **Vasiliki Peppa**: Experimental data collection; manuscript review. **Costas J. Hourdakis**: Experimental data collection; manuscript review. **Pantelis Karaiskos**: Experimental data collection; manuscript review. **Aristea Lekatou**: Data curation. **Evaggelos Pantelis**: Research design; data collection; manuscript writing and review. All authors have read and approved the final version of the manuscript.

## CONFLICT OF INTEREST STATEMENT

The authors declare no conflicts of interest.
